# Ang II Controls the Expression of Mapkap1 by miR-375 and Affects the Function of Islet β Cells

**DOI:** 10.2174/1871530323666230206121715

**Published:** 2023-07-19

**Authors:** Xiuhong Lin, Lin Cheng, Yan Wan, Yuerong Yan, Zhuo Zhang, Xiaohui Li, Jiayun Wu, Xiaoyi Wang, Mingtong Xu

**Affiliations:** 1 Department of Clinical Nutrition, Sun Yat-sen Memorial Hospital, Sun Yat-sen University, No. 107 Yanjiangxi Road, Guangzhou, Guangdong, 510120, People’s Republic of China;; 2 Department of Endocrinology, Sun Yat-sen Memorial Hospital, Sun Yat-sen University, No. 107 Yanjiangxi Road, Guangzhou, Guangdong, 510120, People’s Republic of China, China

**Keywords:** RAS, angiotensin II, miR-375, mapkap1, islet β-cells, phosphorylation

## Abstract

**Background:**

The RAS system is involved in the regulation of islet function, but its regulation remains unclear.

**Objective:**

This study investigates the role of an islet-specific miR-375 in the effect of RAS system on islet β-cells.

**Methods:**

miR-375 mimics and inhibitors were transfected into insulin-secreting MIN6 cells in the presence or absence of RAS component.

**Results:**

Compared to control, in Ang II-treated MIN6 cells, miR-375 mimic transfection results in a decrement in cell viability and Akt-Ser levels (0.739±0.05 *vs.* 0.883±0.06 and 0.40±0.04 *vs*. 0.79±0.04, respectively), while the opposite occurred in miR-375 inhibitor-transfected cells (1.032±0.11 *vs.* 0.883±0.06 and 0.98±0.05 *vs.* 0.79±0.04, respectively, *P*<0.05). Mechanistically, transfection of miR-375 mimics into Ang II-treated MIN6 cells significantly reduced the expression of Mapkap1 protein (0.97±0.15 *vs.* 0.63±0.06, *P*<0.05); while miR-375 inhibitor-transfected cells elevated Mapkap1 expression level (0.35±0.11 *vs*. 0.90±0.05, *P*<0.05), without changes in mRNA expression. Transfection of miR-375 specific inhibitors TSB-Mapkap1 could elevate Mapkap1 (1.62±0.02 *vs.* 0.68±0.01, *P*<0.05), while inhibition of Mapkap1 could significantly reduce the level of Akt-Ser473 phosphorylation (0.60±0.14 *vs.* 1.80±0.27, *P*<0.05).

**Conclusion:**

The effects of Ang II on mouse islet β cells were mediated by miR-375 through miR-375/Mapkap 1 axis. This targeted regulation may occur by affecting Akt phosphorylation of β cells. These results may provide new ideas and a scientific basis for further development of miRNA-targeted islet protection measures.

## 1 INTRODUCTION

According to the Global Diabetes Map (10^th^ edition) released by the International Diabetes Federation (IDF) in December 2021 [1], about 537 million adults (20-79 years old) in the world suffering from diabetes in 2021 (10.5%), and this number is expected to rise to 643 million (11.3%) and 783 million (12.2%) by 2030 and 2045, respectively.

In diabetes patients, islet damage is a key factor influencing the occurrence and development of diabetes [2]. However, the detailed mechanisms of islet damage and repair are not yet understood, and there is a lack of effective measures to protect and reverse islet damage clinically [3]. Recent studies have shown that the insulin signaling pathway plays an important role in maintaining both the number and function of islet β cells [4]. Insulin receptor (IR), insulin receptor substrate (IRS-1, IRS-2), and various protein kinases (PI-3K, PKB, PKC, MAPK, and MAK) can be expressed in pancreatic β cells in both rodents and humans, suggesting the existence of insulin autocrine in β cells [5]. Islet β cells are also the target cells of insulin, and dysfunction of insulin signal transduction in β cells can lead to an impaired number and function of β cells, which is one of the important pathophysiological changes in diabetes [6].

The renin-angiotensin system (RAS) has been widely studied in blood vessels [7], and recently it has been found to play an important role in islet injury and repair [8]. Angiotensin II (Ang II) is the main active substance of RAS, which affects islet function either directly or indirectly by affecting microcirculation blood flow, thus causing islet fibrosis, reducing the proliferation and apoptosis of β cells, and inhibiting insulin biosynthesis and secretion [9, 10]. The angiotensin-converting enzyme (ACE)-Ang II-Angiotensin II type 1 receptor (AT1R) pathway and the ACE2-Ang-(1-7)-Mas receptor (MasR) pathway are antagonistic, and the balance between these two axes determines the ultimate effect of RAS [11-13]. Activation of the ACE-Ang II-AT1R pathway is one of the important pathological mechanisms of diabetes [14, 15], and as such, blocking this pathway can reduce the risk of diabetes [16-17]. However, in-depth mechanistic study on how RAS regulates insulin in islet β cells remains unknown.

MicroRNAs (miRNAs) are a class of endogenous non-coding single-stranded small RNA molecules composed of 21-25 nucleotides that target the mRNA and participate in the regulation of gene expression after transcription [18]. Studies have reported that miRNA is a key regulator of the number and function of β cells [19-23] and is also involved in the expression of the pathogenic effect of RAS [24-27]; it is, thus, a potential therapeutic target for some diseases. Poy *et al.* first discovered and confirmed miR-375 [28] to be an islet-specific miRNA that is necessary for normal pancreatic development, and is involved in the regulation of insulin secretion, β cell development, proliferation, and apoptosis [29-31]. miR-375 can act on phosphoinositide-dependent protein kinase-1 (PDK1) [32-35], a key component in the insulin signaling pathway, to reduce the protein level of PDK1, thereby inhibiting insulin biosynthesis [30, 36]. In addition, miR-375 was also observed to be involved in fatty apoptosis of islet β cells; that is, high expression of miR-375 could make mouse islet secreting cells more sensitive to fatty apoptosis induced by palmitic acid, while inhibition of miR-375 expression resulted in reduced fatty apoptosis induced by palmitic acid [37]. Our preliminary data also suggest that miR-375 was significantly differentially expressed when Ang II acted on pancreatic β cells (Fig. **S1**), indicating that miR-375 may be an important regulator of RAS that affects islet β cells.

Therefore, in this study, we aim to depict the importance of miR-375 in affecting the RAS pathway. By uncovering their mechanistic pathway, we hope to identify the molecular mechanisms of miR-375 in controlling the effect of RAS on the insulin signal of islet β cells.

## 2 MATERIALS AND METHODS

### 2.1 Cell Culture

All reagents (unless noted otherwise) were purchased from Gibco and Corning, USA. MIN6 cells, mouse insulin-secreting cells were derived from transgenic mouse islet-tumor-expressing SV40T antigens [38], and were purchased from the cell bank of Central South University (Changsha, China). MIN6 cells were cultured in DMEM high-glucose medium (25 mmol/L glucose containing 10% fetal bovine serum and 5 μl /L β mercaptoethanol) in an incubator at 37°C, 5% CO_2_, and saturated humidity, under the same conditions as described in a previous study [39] When the cells grew to about 80% confluence, the cells were sub-cultured at a ratio of 1:3 and were passaged once every 3-4 days (Fig. **S2**).

### 2.2 Real-time PCR (RT-PCR)

Real time PCR (RT-PCR) reagent was purchased from TaKaRa, Japan, PCR reaction plate and instrument were purchased from Bio-Rad, USA, primer sequence was purchased from Guangzhou RiboBio Technology Co., LTD. RT-PCR was used for semi-quantitative analysis. Primer sequences for PCR are shown in Table **S1**.

RT-PCR was used to detect miRNA expression levels after Ang II and Ang (1-7) stimulation in MIN6 cells. After the mice insulin-producing MIN6 cells were stimulated using Ang II / Ang (1-7) (both 10^-5^ mol/L, 48 h), the cells were collected, RNA was extracted. RT-PCR was conducted to confirm that miR-375 is regulated by RAS in the insulin signaling pathway of pancreatic islets.

### 2.3 Sanger microRNA Registry Database

To obtain sequence of miR-375 and miR-375 mimics/inhibitors, the mature sequence information of miR-375 (UUUGU UCGUUCGGCUCGCGUGA) was obtained through the Sanger microRNA Registry database, primers, and corresponding mimics/inhibitors (2'-O-methylated antisense oligonucleotides) of miR-375 were designed accordingly.

### 2.4 Transfection of Mimics and Inhibitors for miR-375

To transfect MIN6 cells with mimics and inhibitors of miR-375 by liposome to enhance or inhibit miR-375 levels, LipofectamineTM 2000 (Invitrogen, USA) was used according to manufacturer’s protocol. To obtain miR-375 overexpressed/inhibited MIN6 cells, the cells were inoculated one day before transfection, and a medium without antibiotics was added to each well such that the cell density at the time of transfection could reach 30-50% for transfection. Analogues and inhibitors of miR-375 were diluted with serum-free optimized medium and mixed gently. Transfection reagent was diluted with serum-free optimized medium and incubated at room temperature for 5 min, then gently mixed with the diluted miRNA and cultured at room temperature for 20 min. The resulting mixture was added to the wells containing the cells and culture medium. For the experiment, the mixture was divided into the transfection group, negative control group, and blank control group. The culture plate was placed in an incubator for culturing, and 6-8 h later, the culture plate was replaced with a complete medium containing serum for further induced differentiation.

### 2.5 Annexin V/PI Double Staining Flow Cytometry

To quantify the apoptosis level in MIN6, Annexin V-FITC Apoptosis Kit (with PI, BD, USA) was purchased; while Hoechst 33342 staining was performed for morphological observation and quantitative analysis. The cells were observed and photographed under an inverted fluorescence microscope; excitation light of 200-380 nm and emission light of 420 nm was used. The nuclei of apoptotic cells showed varying degrees of solid shrinkage and hyperchromatic staining. Five horizons were randomly selected from each sample to calculate the rate of apoptosis.

Cultured cells were collected, and 100 μl of Binding Buffer and 10 μl of FITC-labeled Annexin V (20 ug/ml) were added. The solution sat at room temperature in darkness for 30 min, and then 5 μl of PI (50 µg/ml) was added. After 5 min of light avoidance reaction, 400 μl of Binding Buffer was added, which was used for flow cytometry quantitative detection. A tube without Annex V-FITC and PI was used as a negative control. Apoptosis was analyzed according to the calculated apoptosis rate.

### 2.6 Cell Counting Kit-8 (CCK-8)

To determine cell proliferation activity of MIN6 cells, CCK-8 assay (Dojindo Laboratories, Japan) was used. Culture medium containing 10% CCK-8 reagent was then added to each well. The cells were incubated at 37°C and 5% CO_2_ for 3 h, followed by detection on a microplate reader (450 nm wavelength). The optical density (OD) value was proportional to the number of cells, and cell proliferation was indicated by (A450 nm = absorbance value of experimental group - absorbance value of blank group). Cell growth and the proliferation curve were calculated according to the OD value.

### 2.7 Radioimmunoassay

To detect the amount of insulin secreted by MIN6 cells, a 125I insulin radioimmunoassay kit (Beijing North Institute of Biotechnology, China) was used. Insulin standard concentrations were 0, 5, 15, 45, 135, 405μIU/ml, respectively. Insulin quality control serum range were as follows: QL: 22.4 (15.7-29.1); QH: 77.9 (54.5-101.3). With the automatic γ counter to determine the precipitation of each tube radioactive count (cpm), the radioactivity measured by each standard tube as a standard curve, the use of ELISAMATE software (Product Code: 1514353, Version 1.1) to fit the four-parameter equation, the concentration of the sample to be measured can be obtained from the standard curve according to the radioactivity count. The insulin content in the sample was corrected by the protein amount for graph analysis.

### 2.8 Bioinformatics Software and Dual Luciferase Reporting Assay

To predict and validate the target genes that might be regulated by miR-375, bioinformatics software and dual luciferase reporting assay were utilized. Based on the structure and thermodynamics of the binding between the miRNA seed sequence and the 3’UTR end of the target gene, it was used to predict the target genes that miRNA might regulate. The main bioinformatics software and databases used included the following: NCBI nucleic acid sequence database (http://www.ncbi.nlm.nih.gov); MiRTarbase database (http://mirtarbase.mbc.nctu.edu.tw); MicroRNA database miRBase (http://ww w.mirbase.org); Targetscan (http://www.targetscan.org/mm u_61); and MiRanda (http://www.microrna.org).

A Dual-Luciferase Reporter Assay Kit was used to assay the luciferase activity and observe whether miR-375 inhibited the transcriptional activity of the Mapkap1 gene, so as to determine whether miR-375 targeted regulating Mapkap1. Dual-Glo^®^ Luciferase Assay System was purchased from Promega, enzymatic reagents from Fermentas, USA. TIANprep Mini Plasmid Kit, Universal DNA Purification Kit and DH5α receptive cell were purchased from Tiangen Biochemical Technology Co., LTD, China.

For the construction of a Mapkap1 reporter gene vector, primers of the Mapkap1 gene 3’UTR were designed using the Primer 5 software. Restriction site sequences (XhoI and EcoRI) were introduced at both ends, respectively, genomic DNA was extracted, PCR amplification was conducted, and the promoter region of the Mapkap1 gene was cloned. After sequencing was confirmed, the cloned promoter fragments were inserted into the MCS region of the luciferase reporter vector 3.1-Luc (Promega), and the Mapkap1 luciferase reporter gene carrier 3.1-luc- Mapkap1-wt was constructed. Meanwhile, the mutant vector 3.1-luc- Mapkap1-mut was constructed using a similar method, and only the predicted binding site of miR-375AAGCCAU was mutated into GAUGUGC. The second fluorescent report empty carrier, pGL4.10 [Luc^2^], was purchased from Promega.

For dual-reporter assay, the following was carried out.

- WT + Negative Control (NC): Wild-Type Mapkap1 3’UTR, a double luciferase reporter vector of the Mapkap1 gene, was co-transfected with miR-375 mimic NC;
- WT + mimic: The Wild-Type Mapkap1 3’UTR of the Mapkap1 gene was co-transfected with miR-375 mimic;
- Mut + NC: Mutant luciferase reporter vector, Mutant Mapkap1 3’UTR, was co-transfected with miR-375 mimic NC; and
- Mut + mimic: Mutant luciferase reporter vector, Mutant Mapkap1 3’UTR, was co-transfected with miR-375 mimic.

### 2.9 Western Blot Analysis

The reagents and instruments were purchased from Shanghai Sheneng Biotechnology Co., Ltd. (China), Roche (Switzerland), TaKaRa (Japan), Bio-Rad, Amresco and Sigma (USA), *etc*.

### 2.10 Detection of Mapkap1 Protein Level Changes after miR-375 Overexpression or Inhibition

To verify whether Mapkap1 is involved in miR-375-regulated mechanism.

miR-375 overexpression/ expression-inhibition system groups are divided as follows: (i)Con group: control group; (ii)mimic/ inhibitor NC group: transfected with miR-375 mimic/ inhibitor NC; (iii)mimic/ inhibitor group: transfected with miR-375 mimic/ inhibitor. Total RNA was extracted 24 hours after transfection and total protein was extracted 36 hours after transfection. RT-PCR was used to detect the expression of miR-375 and Western blot was used to detect the level of Mapkap1 protein (anti-Mapkap1, Millipore (#05-1044), USA).

### 2.11 Detect changes in Mapkap1 After Ang II Treatment of MIN6 Cells

- Mapkap1 protein levels when treated with different concentrations of Ang II: MIN6 cells were treated with different concentrations of Ang II (0, 10^-8^, 10^-7^, 10^-6^, 10^-5^, and 10^-4^ mol/L) for 36 h.
- Mapkap1 protein levels when treated with different lengths of time: MIN6 cells were treated with 10^-5^ mol/L Ang II for 12 h, 24 h, 36 h, and 48 h.
- Expression of Mapkap1 mRNA when treated with Ang II: Based on the above findings that Ang II affected Mapkap1 protein levels at different concentrations and lengths of time, MIN6 cells were treated with 10^-5^ mol/L Ang II for 24 h, 36 h, and 48 h, and RT-PCR was used to detect Mapkap1 mRNA expression.

### 2.12 Detect Mapkap1 Protein level in MIN6 Cells Transfected with TSB-Mapkap1 and Treated with 10-5 mol/L Ang II

To investigate whether Ang II affect islet β cells by targeting Mapkap1 regulation through miR-375.

TSB-Mapkap1 (microRNA target site Blocker), a specific inhibitor of miR-375, was designed for the corresponding binding site sequence of miR-375 and the Mapkap1 3’UTR. The phenotypic changes obtained by commonly used microRNA inhibitors were the result of the up-regulation of multiple targets. To further test this, the following three test groups were designated in MIN6 cells: (i) control group (Con); (ii) NC group (NC): transfected TSB-Mapkap1 NC; and (iii) TSB-Mapkap1 group: transfected with TSB-Mapkap1. 36 h later, Mapkap1 protein levels in the cells were detected by Western blot, apoptosis rate was detected by Annexin V/PI staining, and insulin secretion was detected by radioimmunoassay.

### 2.13 Assessing Cellular Pathway of Mapkap1

We first silenced the Mapkap1 mRNA through siRNA interference. Three specific siRNAs (Mapkap1-Si-1, Mapkap1-Si-2, and Mapkap1-Si-3) were designed for the Mapkap1 sequence (Guangzhou RiboBio Technology Co., Ltd., China). After 36 h of transfection in MIN6 cells, Mapkap1 protein levels were detected to obtain the best siRNA sequence for silencing. At the same time, levels of protein associated with insulin signaling pathway like Akt (Cell Signaling #4691, USA), Akt-Ser473 (Cell signaling #4058, USA), Caspase-3 (Cell Signaling #9662, USA), cleaved caspase-3 (Cell signaling #9664, USA) and IRβ-Tyr (Santa cruz #Tyr1163/1164, UK) were also detected by Western blot. The UVidoc gel image analysis system (Biometra, Germany) used photographs and band density scans to calculate the ratio of the targeted phosphorylated protein band to the corresponding total protein as the relative level of protein phosphorylation.

Two test groups were designated: (i) NC group: MapKAP1-SiRNA NC was transfected; and (ii) MapKAP1-Si group: MAPKAP1-Si-3 was transfected (the best sequence according to the above experiment).

### 2.14 Statistical Analysis

The statistical software package SPSS 19.0 was used for data analysis. The experimental results are measurement data expressed as mean ± standard deviation (x ± s). All data were tested for normality and homogeneity of variance. Two independent sample t-tests were used for comparing two groups, one-way ANOVA was used for comparing multiple groups, and the least significant difference LSD-T test was used for pairwise comparison between multiple groups. *P* < 0.05 was regarded as statistically significant. Statistical graphs were created using GraphPad Prism 6.0 and were annotated and integrated using Adobe Photoshop CS 6.0 and Adobe Illustrator CS 6.0.

## 3 RESULTS

### 3.1 Changes of miR-375 Expression When RAS Acts on Islets

To detect cellular miR-375 expression changes in mouse insulin-secreting cell MIN6 cells under RAS, we used RT-PCR. After Ang II treatment, expression levels of miR-375 significantly increased in comparison with the control group (3.75±0.008 *vs*. 1, *P* < 0.05) (Fig **S1**). Contrastingly, after Ang (1-7) treatment on MIN6 cells, the expression of miR-375 appeared to be up-regulated, but the difference was not considered statistically significant (1.92±0.023 *vs*. 1, *P* > 0.05) (Fig. **S1**).

### 3.2 Expression of miR-375 in MIN6 Cells

**Fig. (1):**
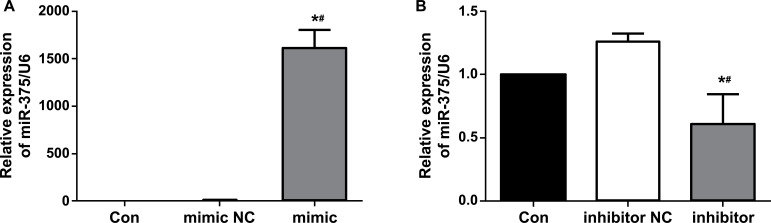
Expression changes of miR-375 after transfection of mimic and inhibitor in MIN6 cells: miR-375 expression was significantly increased after transfection with miR-375 mimic **(A)**, while significantly decreased after transfection with miR-375 inhibitor **(B)**. Real-time PCR confirmed that miR-375 mRNA was overexpressed or inhibited, indicating that the modeling was successful. **Abbreviations:** con: control; NC: normal control; *P<0.05 vs. blank control, ^#^P<0.05 vs. NC.

To establish the overexpression/ inhibition model of miR-375 in mouse insulin-secreting cells, miR-375 mimic and inhibitor were transfected to MIN6 cells with LipofectamineTM 2000. After 24 h, cells were collected to extract total RNA and detect the changes in the expression of miR-375. The results showed that miR-375 expression was significantly increased after transfection with miR-375 mimic (1616.33 ± 191.17 *vs*. 1, *P* < 0.05) (Fig. **1A**), while it was significantly decreased after transfection with miR-375 inhibitor (0.61 ± 0.24 *vs*. 1, *P* < 0.05) (Fig. **1B**), indicating that the modeling was successful.

### 3.3 Changes of Mouse Islet β Cells under Ang II after the Expression of miR-375 was Changed

**Fig. (2):**
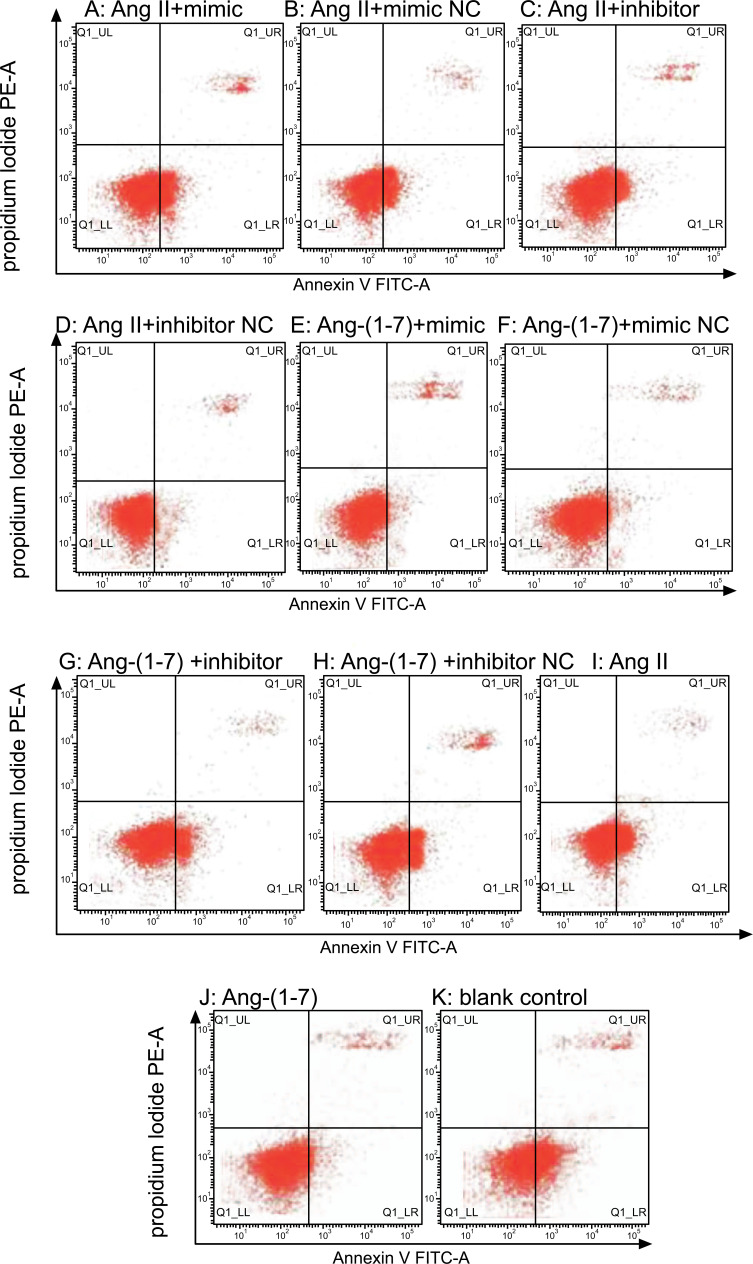
Cell apoptosis detected by Flow cytometry (**A**-**K**). Results showed that compared to control, in Ang II-treated MIN6 cells, miR-375 mimic transfection increased cell apoptosis (10.20±3.54 *vs*. 8.92±1.16, *P* < 0.05), while the opposite occurred in miR-375 inhibitor-transfected cells (7.46±1.72 *vs*. 8.92±1.16, *P* < 0.05). Ang-(1-7) did not significantly reduce cell apoptosis compared with the control group (7.12±1.64 *vs*. 8.06±0.91, *P* > 0.05), but transfected with miR-375 mimic significantly increased cell apoptosis compared with the control group (8.72±2.23 *vs*. 7.12±1.64, *P* < 0.05). *Ang II: angiotensin II, Ang-(1-7): angiotensin-(1-7), NC: normal control.*

**Fig. (3):**
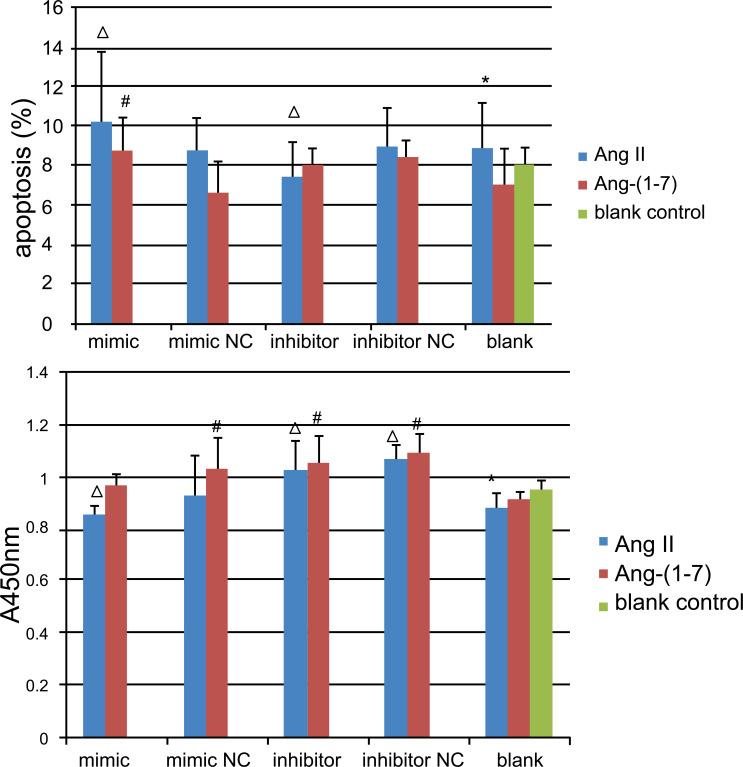
Histogram of cell apoptosis (**A**): data are the same as in legend of Fig. **2**. Histogram of cell proliferation as measured by CCK8 (**B**). Cell viability was significantly decreased after transfection with miR-375 mimic (0.739 ± 0.05 *v*s. 0.883 ± 0.06, *P* < 0.05), while increased after transfection with miR-375 inhibitor (1.032 ± 0.11 *vs*. 0.883 ± 0.06, *P* < 0.05) compared with the control group under the effect of Ang II. Ang-(1-7) did not significantly increase the cell viability compared with the control group (0.915 ± 0.03 *vs*. 0.956 ± 0.03, *P* > 0.05), but the cell viability was significantly increased after transfection with miR-375 inhibitor compared with the control group (1.062 ± 0.10 *vs*. 0.915 ± 0.03, *P* < 0.05). Ang II: angiotensin II, Ang-(1-7): angiotensin-(1-7), NC: normal control. *P<0.05 vs. control group; ΔP< 0.05 vs. Ang II blank control group; ^#^P< 0.05 vs. Ang-(1-7) blank control group.

To observe the role of miR-375 in the regulation of Ang II on the apoptosis of islet β cells, we used Annexin V/PI staining and the results showed that apoptosis of cells transfected with miR-375 mimic under the effect of Ang II was significantly increased compared with the control group, while cell viability after transfection with miR-375 inhibitor was not statistically different compared with the control group. Ang (1-7) did not significantly reduce cell apoptosis compared with the control group, but transfection with an miR-375 mimic significantly increased cell apoptosis compared with the control group (Table **1**, Figs. **2A**-**K**, Fig. **3A**).

To understand the role of miR-375 in the regulation of Ang II on the proliferation of islet β cells, CCK-8 results showed that cell viability was significantly decreased after transfection with miR-375 mimic, but increased after transfection with miR-375 inhibitor, compared with the control group under the effect of Ang II. However, Ang-(1-7) did not significantly increase cell viability compared with the control group, while cell viability was significantly increased after transfection with miR-375 inhibitor compared with the control group (Table **1**, Fig. **3B**).

**Fig. (4):**
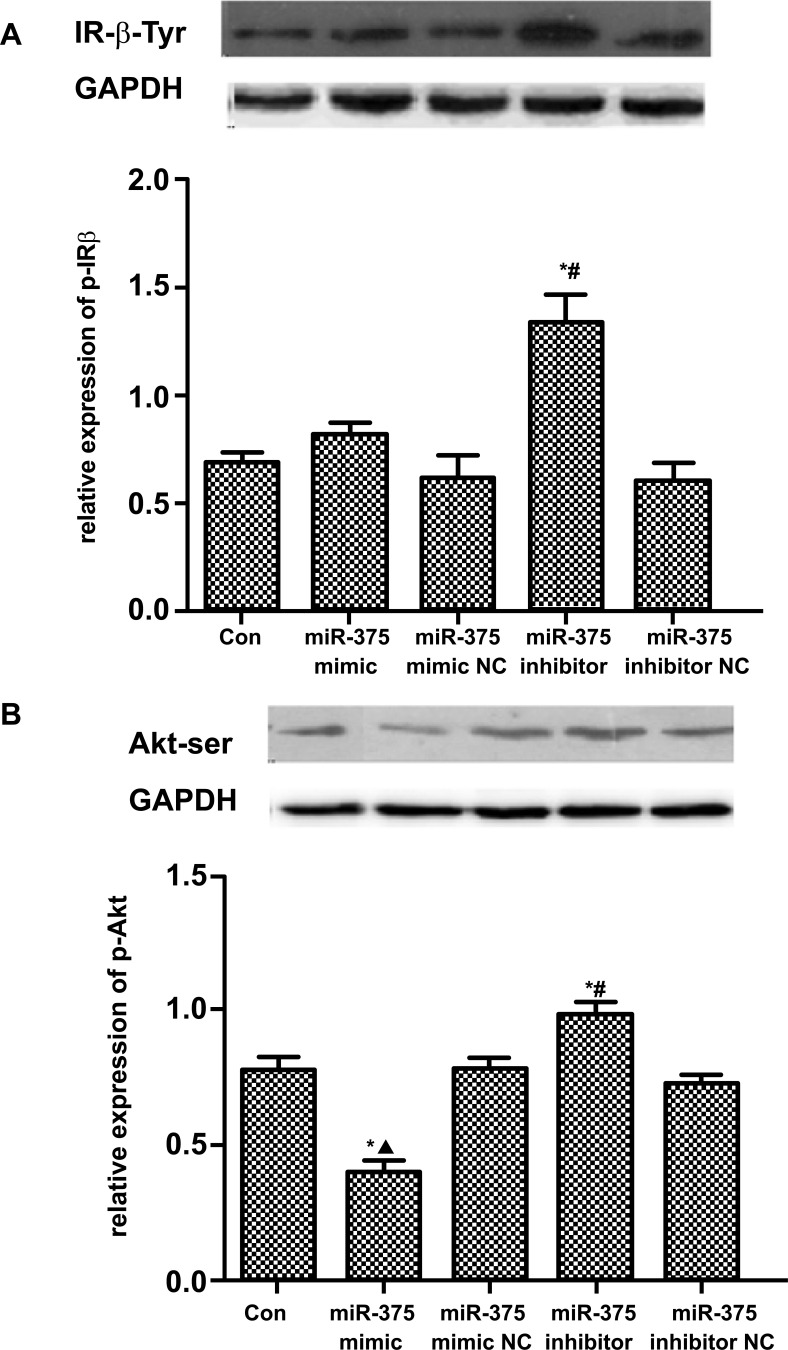
Western blot for detecting phosphorylation levels of IR-β (**A**) and Akt (**B**). Akt-Ser levels were further decreased after transfection with miR-375 mimic (0.40 ± 0.04 *vs*. 0.79 ± 0.04, *P* < 0.05). After transfection with miR-375 inhibitor, IR-β-Tyr and Akt-ser levels were both increased (1.35 ± 0.12 *vs*. 0.69 ± 0.05 and 0.98 ± 0.05 *vs*. 0.79 ± 0.04, both *P* < 0.05). **Abbreviations: ***Con: control; NC: normal control.* Note: *P< 0.05 vs. control group; ^#^P< 0.05 vs. miR-375 inhibitor NC; ▲P< 0.05 vs. miR-375 mimic NC. ^#^P < 0.05 vs. Mut+NC. (BCDE) *P < 0.05 vs. Con; (BC) ^#^P < 0.05 vs. NC; (D) ^#^P<0.05 vs. 10^-6^ AngII; (E) ^#^P<0.05 vs. 24h AngII; *▲P<0.05 vs. 36h AngII.*

To explore the role of miR-375 in the regulation of Ang II on insulin signaling of islet β cells, western blot was used to detect the expressions of IRβ-Tyr and Akt-ser in the insulin signaling pathway of MIN6 cells. Akt-Ser levels were found to be further decreased after transfection with miR-375 mimic in Ang II-treated MIN6 cells (0.40 ± 0.04 *vs*. 0.79 ± 0.04, *P* < 0.05). While after transfection with miR-375 inhibitor, tyrosine phosphorylated IR-β (IR-β-Tyr) and Akt-Ser levels were increased (1.35 ± 0.12 *vs*. 0.69 ± 0.05 and 0.98 ± 0.05 *vs.* 0.79 ± 0.04, both *P* < 0.05) (Table **2**, Fig. **4**).

### 3.4 Bioinformatics Predictions of Molecular Mechanisms of miR-375

#### 3.4.1 Prediction of Target Genes Directly Regulated by miR-375

More than 100 miR-375 target genes were predicted by bioinformatics software, and Mapkap1 gene was one of them. There is a binding site of miR-375 on Mapkap1 3'UTR [GAACAAA (2355-2361), Position 313-319 of Mapkap1 3' UTR)]. The predicted results of Targetscan online software and miRanda online software are consistent (Fig. **S3**). Mapkap1 is an important component of mTORC2 [40], and mTORC2-Akt plays a certain role in regulating the quantity and function of pancreatic β cells [41]. Combined with the reported effects of miR-375, the physiological effects of these target genes, and the possible molecular mechanisms involved, this study selected Mapkap1 as the target gene that miR-375 may directly regulate for further study.

**Fig. 5:**
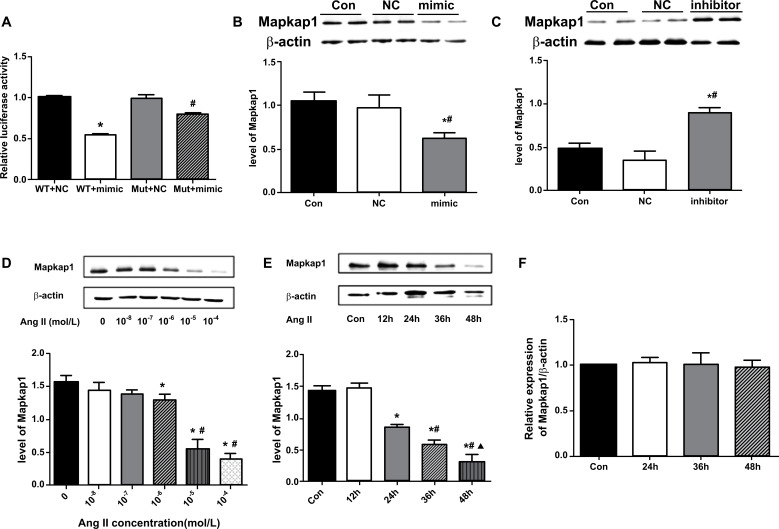
(**A**) Experiment of dual-luciferase reporter gene. miR-375 mimic significantly down-regulated the reported fluorescence of Mapkap1 wild-type vector, which recovered after corresponding binding site mutation. These results suggest that Mapkap1 may be the target gene directly regulated by miR-375. Effect of miR-375 on Mapkap1 protein expression in MIN6 cells with Western blot: (**B**) Mapkap1 protein level significantly decreased 36 h after transfection of miR-375 mimic, (**C**) while increased after transfection with miR-375 inhibitor. Effects of Ang II on target genes of miR-375 in MIN6 cells: changes of Mapkap1 protein levels in islet β cells (**D**) treated with different concentrations of Ang II and (**E**) after Ang II treatment for different times. (**F**) Changes of Mapkap1 mRNA levels in MIN6 cells treated with Ang II at different time: The results showed that there was no significant difference in the expression level of Mapkap1 mRNA among all groups (*P* >0.05). **Abbreviations:** WT: wild type; Mut: Mutant; NC: normal control; RFU: Relative Fluorescence Unit; Con: control; Ang II: angiotensin II. (A)*P < 0.05 vs. WT+NC; ^#^P < 0.05 vs. Mut+NC. (BCDE) *P < 0.05 vs. Con; **(B-C)** ^#^P < 0.05 vs. NC; **(D)** ^#^P<0.05 vs. 10^-6^ AngⅡ; **(E)** #P<0.05 vs. 24h AngⅡ; ▲P<0.05 vs. 36h AngⅡ.

In order to verify whether miR-375 directly binds to the 3’ UTR of Mapkap1, the double luciferase reporter gene experiment was conducted, and the interaction between miRNA and target genes was confirmed by down-regulating the relative fluorescence value of reporter genes. The results showed that miR-375 mimic significantly down-regulated the reported fluorescence of the Mapkap1 wild-type vector (0.55 ± 0.15 *vs*. 1 ± 0.02, *P* < 0.05), which recovered after corresponding binding site mutation (0.80 ± 0.01 *vs*. 0.55 ± 0.15, *P* < 0.05) (Fig. **5A**). These results suggested that by binding to the 3’UTR binding site of Mapkap1, miR-375 inhibits the transcription of Mapkap1 and negatively regulates the expression of Mapkap1, and Mapkap1 is the target gene directly regulated by miR-375.

#### 3.4.2 Changes of Mapkap1 Protein Level after Overexpression or Inhibition of miR-375 in MIN6 Cells

To detect the effect of miR-375 on Mapkap1 protein expression in MIN6 cells, we used western blot and the results showed that the Mapkap1 protein level decreased significantly after transfection with miR-375 mimic (0.97 ± 0.15 *vs*. 0.63 ± 0.06, *P* < 0.05) (Fig. **5B**), but increased after transfection with miR-375 inhibitor (0.35 ± 0.11 *vs*. 0.90 ± 0.05, *P* < 0.05) (Fig. **5C**).

#### 3.4.3 Changes of Mapkap1 Protein Level after Ang II Treatment of Islet β Cells

To study the effect of Ang II on miR-375 target gene Mapkap1, MIN6 cells were treated with different concentrations of Ang II (0, 10^-8^, 10^-7^, 10^-6^, 10^-5^, 10^-4^ mol/L) for 36 h. Western blot results showed that the Mapkap1 protein level of MIN6 cells treated with 10^-6^, 10^-5^, and 10^-4^ mol/L Ang II were significantly lower than those of the blank group, 10-8, and 10^-7^Ang II groups (1.29 ± 0.09, 0.55 ± 0.15, 0.40 ± 0.08 *vs*. 1.56 ± 0.11, 1.44 ± 0.12, 1.39 ± 0.06, respectively, *P* < 0.05). Expression of the Mapkap1 protein in the 10- and 10^-4^Ang II groups was further decreased than that in the 10^-6^Ang II groups (0.55 ± 0.15, 0.40 ± 0.08 *vs*. 1.29 ± 0.09, *P* < 0.05). No statistical difference was found between the 10^-5^ and 10^-4^Ang II groups (0.55 ± 0.15 *vs*. 0.40 ± 0.08, *P* > 0.05) (Fig. **5D**). These results suggested that, to some extent, Ang II could down-regulate the Mapkap1 protein level in MIN6 cells in a concentration-dependent manner.

In order to investigate whether the effect of Ang II on Mapkap1 is time-dependent, MIN6 cells were treated with 10^-5^ mol/L Ang II for 12 h, 24 h, 36 h, and 48 h. Western blot results showed no statistical difference between the 12-h Ang II group and the blank group (1.48 ± 0.08 *vs*. 1.42 ± 0.09, *P* > 0.05). However, the level of Mapkap1 protein in the Ang II group was significantly lower than that in the blank group at 24 h, 36 h, and 48 h (0.86 ± 0.04, 0.58 ± 0.08, 0.31 ± 0.12 *vs*. 1.42 ± 0.09, *P* < 0.05). The expression of Mapkap1 protein in the 36-h and 48-h Ang II groups was further decreased than that in the 24-h Ang II group (0.58 ± 0.08, 0.31 ± 0.12 *vs*. 0.86 ± 0.04, *P* < 0.05); likewise, expression in the 48-h Ang II group was further decreased than that in the 36-h Ang II group (0.31 ± 0.12 *vs*. 0.58 ± 0.08, *P* < 0.05) (Fig. **5E**). These results suggest that Ang II down-regulated Mapkap1 in MIN6 cells in a dose - and time-dependent manner.

In order to further investigate the molecular level of Ang II inhibition of Mapkap1, based on the findings that Ang II treatment could affect Mapkap1 protein levels at different concentrations and lengths of time, the expression of Mapkap1 mRNA was detected by real-time PCR in MIN6 cells treated with 10^-5^ mol/L Ang II for 24 h, 36 h, and 48 h. The results showed no significant difference in the expression level of Mapkap1 mRNA among all the groups (1.03 ± 0.06, 1.01 ± 0.13, and 0.98 ± 0.07, respectively, *P* > 0.05) (Fig. **5F**), which showed no change trend compared with the non-intervention group.

#### 3.4.4 Functional Changes of MIN6 Cells After Ang II Target-Regulating Mapkap1 through miR-375

**Fig. (6):**
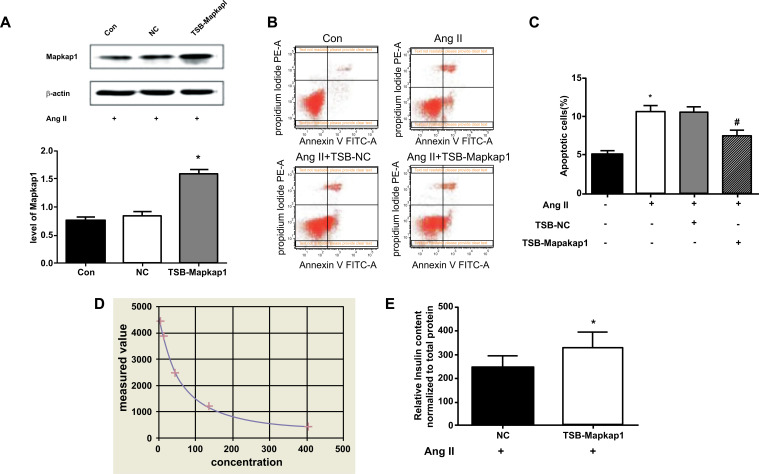
Ang II affected MIN6 cells by targeting Mapkap1 regulation by miR-375: (**A**) on the change of Mapkap1 protein: level of Mapkap1 protein in TSB-MAPKAP1 group was significantly higher than that in Con group (*P* < 0.05), indicating that transfection of MapKAP1-TSB inhibited the negative regulation of miR-375 on target gene Mapkap1; (**B, C**) on the change of cell apoptosis: compared with Con group, the apoptosis level of Ang II group was significantly increased (*P* < 0.05); transfection of MAPKAP1-TSB statistically reduced the apoptosis induced by Ang II (*P* < 0.05); (**D, E**) on the change of insulin secretion: ^125^I standard curve of insulin radioimmunoassay fits well; Mapkap1-TSB increased insulin secretion of Ang II treated cells compared with NC group (*P* < 0.05). **Abbreviations:** *con: control; NC: normal control; TSB: target site blocker; AngII: angiotensinII.* ***Note:*** *P < 0.05 vs. Con; ^#^*P* < 0.05 *vs.* AngII.

In order to investigate the effect of Ang II on islet β cells through the targeted regulation of Mapkap1 by miR-375, we transfected TSB-Mapkap1 to MIN6 cells, which specifically inhibited the negative regulation of miR-375 on the target gene Mapkap1. MIN6 cells were simultaneously transfected with TSB-Mapkap1 and processed with 10^-5^ mol/L Ang II. Thirty-six hours later, the *level* of Mapkap1 protein in the TSB-Mapkap1 group was significantly higher than that in the control group (1.62 ± 0.02 *vs*. 0.68 ± 0.01, *P* < 0.05) (Fig. **6A**), which indicated that transfection of TSB-Mapkap1 inhibited the negative regulation of miR-375 on the target gene Mapkap1 caused by Ang II.

To investigate the effect of TSB-Mapkap1 treatment on apoptosis induced by Ang II, MIN6 cells were transfected with TSB-Mapkap1 and treated with 10^-5^ mol/L Ang II. Thirty-six hours later, the apoptosis rate was detected by Annexin V/PI staining. The results showed that compared with the Con group, the apoptosis level in the Ang II group was significantly increased, and the difference was statistically significant (*P* < 0.05). Transfection of TSB-Mapkap1 reduced the apoptosis induced by Ang II, and the difference was statistically significant (*P*< 0.05) (Figs. **6B, C**).

In addition, to investigate the effect of TSB-Mapkap1 treatment on Ang II induced insulin secretion in MIN6 cells, an insulin radioimmunoassay was used and showed that the quality control serum was within the target value range, QL:21.04 (15.7-29.1) and QH:72.26 (54.5-101.3), the standard curve fits well (Fig. **6D**) and the following two equations were derived: the four-parameter logarithm equation, Y = 96.5985 + (4788.33-96.5985) / (1 + (0.020708*X)^1.17389; the four-parameter logarithm regression coefficient: R = 0.999474. MIN6 cells were transfected with TSB-Mapkap1 and treated with 10^-5^ mol/L Ang II. Insulin secretion was then detected by radioimmunoassay 36 hours later. The results showed that TSB-Mapkap1 increased insulin secretion of Ang II-treated cells compared with the NC group (242.11 ± 52.33 μIU/ml∙ug/μl protein *vs*. 326.12 ± 69.53 μIU/ml∙ug/μl protein, *P* < 0.05) (Fig. **6E**).

#### 3.4.5 Functional Changes in Mice Islet β Cells Induced by Mapkap1 Through miR-375 Regulation in Akt Pathway-Dependent Manner

**Fig. (7):**
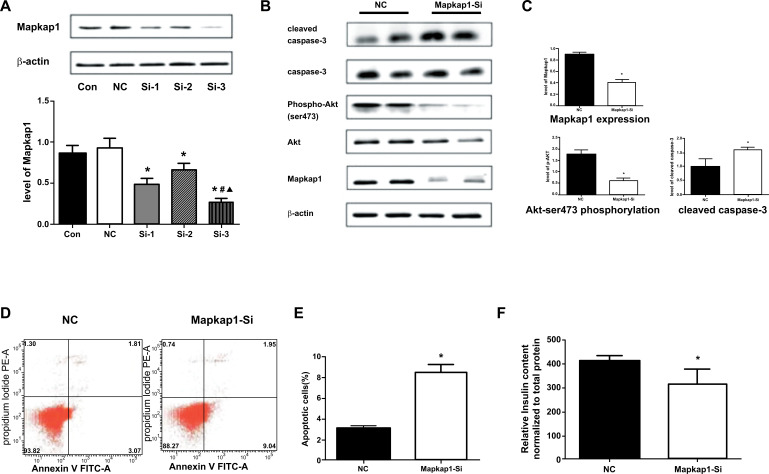
Mapkap1 plays a role through Akt in MIN6 cells: (**A**) SiRNA that can effectively interfere with Mapkap1 protein expression was screened in MIN6 cells: the protein level of Mapkap1 in Si-3 group was lower than that in Con group, Si-1 group and Si-2 group (all *P* < 0.05). (**B, C**) After Mapkap1 expression was inhibited, Akt-Ser473 phosphorylation was significantly reduced and the target molecular activation type of apoptotic molecules cleaved caspase-3 levels were upregulated in MIN6 cells (*P* < 0.05). (**D, E**) Apoptosis rate of MIN6 cells after siRNA interference with Mapkap1 expression was significantly increased (*P* < 0.05). (**F**) Insulin secretion of MIN6 cells was significantly decreased after siRNA interference with Mapkap1 expression (*P* < 0.05). **Abbreviations:** Con: control; NC: normal control; *P<0.05 vs. Con (A)/NC (BCDEF) ^#^P<0.05 vs. Si-1;▲P<0.05 vs. Si-2.

To investigate whether Mapkap1 plays a role in β cells through Akt, three specific siRNAs (Si-1, Si-2, and Si-3) were designed for Mapkap1 sequence to transfect MIN6 cells. Western blot showed that the Mapkap1 protein level in Si-3 group was lower than that in Con, Si-1 and Si-2 group (*P* all < 0.05) (Fig. **7A**), indicating that Si-3 can effectively interfere with Mapkap1 expression, and the interference effect is the best.

To observe the phosphorylation levels of Akt-ser473 and cleaved caspase-3 levels after inhibition of Mapkap1 expression, MIN6 cells were transfected with siRNA fragment Si-3, which could effectively interfere with Mapkap1 protein levels (0.41 ± 0.07 *vs*. 0.93 ± 0.07, *P* < 0.05). The western blot results showed that compared with the NC group, inhibition of Mapkap1 expression significantly reduced Akt-Ser473 phosphorylation levels (0.60 ± 0.14 *vs*. 1.80 ± 0.27, *P* < 0.05) and up-regulated apoptotic target caspase-3 (1.59 ± 0.12 *vs*. 0.99 ± 0.42, *P* < 0.05) (Figs. **7B, C**).

To study the effect of siRNA interfering expression of Mapkap1 protein on apoptosis, MIN6 cells were transfected with siRNA fragment Si-3, and Annexin V/PI staining results showed that the rate of apoptosis in Mapkap1-inhibited cells was significantly increased compared with the NC group (*P* < 0.05) (Figs. **7D, E**).

To explore the effect of siRNA interfering expression of Mapkap1 protein on insulin secretion, MIN6 cells were transfected with siRNA fragment Si-3, and radioimmunoassay results showed that compared with the NC group, insulin secretion of Mapkap1 inhibited cells was significantly decreased (316.89 ± 61.28 μIU/ml *vs*. 412.92 ± 22.09 μIU/ml, *P* < 0.05) (Fig. **7F**).

## 4 DISCUSSION

Islet injury is the core mechanism of diabetes, in which Ang II plays an important role with unclear mechanisms. Our research shows that: (1) Ang II can inhibit the proliferation of pancreatic β cells, increase apoptosis and block insulin signaling by up-regulating the expression of miR-375; (2) Mapkap1 is a target gene of miR-375; as well as (3) Ang II regulates the apoptosis and insulin secretion of insulin-secreting cells probably through the mTORC2-Akt pathway by targeting Mapkap1 expression through miR-375.

In the current study, Ang II increased the level of miR-375 in mouse pancreatic β cell MIN6 cells. It has been reported that miRNA induces the pathogenic effect of RAS and mediates the pathophysiological role of Ang II [26, 42-44]. In vascular smooth muscle cells (VSMC), Ang II has been found to up-regulate the expression of miR-130a and decrease the level of GAX, the target gene of miR-130a, in a concentration- and time-dependent manner, thus promoting cell proliferation [45]. In human umbilical vein endothelial cells (HUVEC), Ang II reportedly down-regulated miR-590-5p, which induced up-regulation of lectin-like oxidized low-density lipoprotein receptor 1 (LOX-1), which increased reactive oxygen species and induced apoptosis [46]. In cardiomyocytes, miR-375 inhibitors inhibited Ang II-induced myocardial hypertrophy by promoting lactate dehydrogenase B (LDHB) expression [47]. It has also been reported that miR-375 mediates the inhibition of β cell proliferation [48], promotes apoptosis [49], and affects the insulin signaling pathway [50]. However, while RAS is significantly activated in T2DM, whether miR-375 is involved as an intermediate substance in Ang II-induced β cell damage and insulin signaling pathway abnormalities has not been reported. Therefore, this study provides evidence that Ang II-induced decrease in proliferation and increase in apoptosis of islet β cells were partially mediated by miR-375, and overexpression of miR-375 could further reduce Akt Serine phosphorylation in islet β cells, while inhibiting expression of miR-375 could reverse the resistance effect on insulin signaling by Ang II. These results suggested that the effect of Ang II on the function in islet β cells was mediated by miR-375, which has not been reported in previous studies.

The mechanism of Ang II mediated by miR-375 in the function of islet β cells was further investigated. And the prediction with bioinformatics software, verification with double luciferase reporter assay and cell transfection confirmed that Mapkap1 was the target gene of miR-375, which has not been found before. MiR-375 has a variety of target genes, and the related target genes in the study of biological function of islet β cells are mainly divided into three categories [30]: Genes related to pancreatic development (such as Sox17, Sox9., *etc.*) [51], cell proliferation (such as Cav1, Id3., *etc.*) [52] and insulin secretion (including MTPN [28] and PDK1 [36]). Mitogen-activated protein kinase associated protein 1 (Mapkap1), also known as stress activated protein kinase interaction protein 1 (Sin1), was widely expressed among multiple tissues and organs. Mapkap1 is an important subunit of mTORC2 and very important to the functionality of mTORC2 [40, 53]. Multiple studies have confirmed that Mapkap1 is involved in mTORC2 regulation of Akt-Ser473 [54-57], PKC [58] and the activity of SGK1 by maintaining the stability of mTORC2 [59]. mTORC2 is a key kinase in the phosphorylation of Akt-Ser473, which is essential for the complete activation of Akt [60]. Akt plays a role in inhibiting apoptosis, promoting proliferation and regulating metabolism in cells [61, 62] and is one of the key molecules that regulate islet β cell volume and insulin secretion through a variety of pathways [63, 64]. Activation of Akt kinase can phosphorylate many substrates, which is closely related to the occurrence and development of diabetes [65-67].

As previously shown, the effect of Ang II on the function of islet β cells is mediated by miR-375, and Mapkap1 is the target gene directly regulated by miR-375, does Mapkap1 mediate the involvement of miR-375 in the effect of Ang II on β cells, and *via* the Akt pathway? To solve these two problems, in our study, MIN6 cells were treated with Ang II at different concentrations and different times. The results showed that miR-375 increased, while Mapkap1 protein decreased without change in Mapkap1 mRNA, suggesting that miR-375 may be involved in the negative regulation of Mapkap1 by Ang II through post-transcriptional inhibition. Further transfection of MIN6 cells with TSB-Mapkap1, a specific inhibitor of miR-375, showed that Ang II could affect apoptosis and insulin secretion of MIN6 cells by targeting miR-375 to regulate Mapkap1. In addition, siRNA of Mapkap1 was transfected into MIN6 cells. The results showed that inhibition of Mapkap1 could simulate the effect of Ang II on apoptosis and insulin secretion of MIN6 cells, and the phosphorylation of Akt-Ser473 was decreased, suggesting that Mapkap1 could affect MIN6 cells through Akt. In the study of miR-7a, Liu *et al.* also made similar findings on the regulation of signal pathway of insulin secreting cells by Mapkap1 [68]. In view of the fact that Mapkap1 is an important component of mTORC2 and mTORC2 is involved in regulating the biological function of islet β cells by affecting the phosphorylation of Akt [41, 69, 70], we speculate that miR-375 targets Mapkap1-mediated Ang II to affect islet β cells, and the mechanism may be related to mTORC2-Akt. The specific mechanism still needs further study.

## CONCLUSION

In conclusion, results of this study showed that miR-375 mediates the adverse effects of Ang II on islet β cells, and miR-375 influences Ang II to down-regulate the phosphorylation of Akt-Ser473 in MIN6 cells, therefore regulating the Akt pathway. These findings help to reveal the mechanism of RAS damage to islet β cells, establish some experimental basis for the pathogenesis of diabetes, and provide new ideas for its prevention and treatment.

## AUTHORS’ CONTRIBUTIONS

X. Lin and L. Cheng performed experiments, analyzed data, prepared figures, interpreted results of experiments, and drafted the paper in consultation with M. Xu and X. Wang, who critically corrected the manuscript. Y. Wan, Y. Yan, and Z. Zhang performed experiments, analyzed data, and edited and revised the manuscript. X. Li and J. Wu assisted in conducting experiments and analyzing data. The final version of this manuscript was reviewed and approved by all authors.

## Figures and Tables

**Table 1:** The regulatory role of miR-375 in RAS affecting MIN6 cells (x ± s).

**Group**		**Apoptosis Rate**	**A450 (nm)**
Ang II	mimic mimic NC inhibitor inhibitor NC	10.20 ± 3.54^Δ^ 8.80 ± 1.60 7.46 ± 1.72^Δ^ 9.00 ± 2.08	0.862 ± 0.03^Δ^ 0.934 ± 0.15 1.032 ± 0.11^Δ^ 1.073 ± 0.05^Δ^
And-(1-7)	mimic mimic NC inhibitor inhibitor NC	8.72 ± 2.23^#^ 6.60 ± 1.95 8.00 ± 0.98 8.53 ± 0.91	0.971 ± 0.04 1.033 ± 0.12^#^ 1.062 ± 0.1^#^ 1.095 ± 0.07^#^
Blank Control	AngII Ang-(1-7) blank	8.92 ± 1.16* 7.12 ± 1.64 8.06 ± 0.91	0.883 ± 0.06* 0.915 ± 0.03 0.956 ± 0.03

**Abbreviations:** RAS: renin-angiotensin system, Ang II: angiotensin II, Ang-(1-7): angiotensin-(1-7), NC: normal control.*P < 0.05 *vs.* blank, ^Δ^*P* < 0.05 *vs.* Ang II blank control, ^#^*P* < 0.05 *vs.* Ang-(1-7) blank control.

**Table 2:** Western blot detected phosphorylation levels of IR-β and Akt in MIN6 cells (x ± s).

**Group**	**Relative Protein Expression Level**	
	**IR-β-Tyr/GAPDH**	**Akt-Ser/GAPDH**
Con	0.69 ± 0.05	0.79 ± 0.04
miR-375 mimic	0.82 ± 0.05	0.40 ± 0.04^*▲^
miR-375 mimic NC	0.62 ± 0.10	0.79 ± 0.05
miR-375 inhibitor	1.35 ± 0.12^*#^	0.98 ± 0.05^*#^
miR-375 inhibitor NC	0.61 ± 0.09	0.73 ± 0.03

**Abbreviations:** Con: control; NC: normal control; IR-β-Tyr: tyrosine phosphorylated insulin receptor β subunit, Akt-Ser: Serine phosphorylation of protein kinase. *P< 0.05 *vs.* control group; ^#^*P*< 0.05 *vs.* miR-375 inhibitor NC; ^▲^*P*< 0.05 *vs.* miR-375 mimic NC.

## Data Availability

The datasets analyzed during the current study are not publicly available due to consideration for intellectual property, a number of ongoing active collaborations, and to continuing analyses by the study investigators, but may be available from the corresponding author upon reasonable request.

## LIST OF ABBREVIATIONS

Akt-Ser: Serine Phosphorylation of Protein Kinase
Ang II: Angiotensin II
Ang-(1-7): Angiotensin-(1-7)
HUVEC: Human Umbilical Vein Endothelial Cells
IR-β-Tyr: Tyrosine Phosphorylated Insulin Receptor β Subunit
LDHB: Lactate Dehydrogenase B
Mapkap1: Mitogen-Activated Protein Kinase Associated Protein 1 Gene
MIN6: Mouse Islet *β* Cells
MiRNA: MicroRNA
mTORC2: Mammalian Target Rapamycin Complex 2
NC: Negative Control
RAS: Renin-Angiotensin System
Sin1: Stress-Activated Protein kinAse Interacting Protein 1 Gene
TSB-Mapkap1: miR-375 Specific Inhibitors Designed for the Corresponding Binding Site Sequence of miR-375 and Mapkap1
VSMC: Vascular Smooth Muscle Cells

## References

[1] Ogurtsova K., Guariguata L., Barengo N.C., Ruiz P.L.D., Sacre J.W., Karuranga S., Sun H., Boyko E.J., Magliano D.J. (2022). IDF diabetes Atlas: Global estimates of undiagnosed diabetes in adults for 2021.. Diabetes Res. Clin. Pract..

[2] Joglekar M.V., Parekh V.S., Hardikar A.A. (2011). Islet-specific microRNAs in pancreas development, regeneration and diabetes.. Indian J. Exp. Biol..

[3] Walker J.T., Saunders D.C., Brissova M., Powers A.C. (2021). The human islet: mini-organ with mega-impact.. Endocr. Rev..

[4] Elghazi L., Bernal-Mizrachi E. (2009). Akt and PTEN: β-cell mass and pancreas plasticity.. Trends Endocrinol. Metab..

[5] Assmann A., Hinault C., Kulkarni R.N. (2009). Growth factor control of pancreatic islet regeneration and function.. Pediatr. Diabetes.

[6] Muller D., Huang G.C., Amiel S., Jones P.M., Persaud S.J. (2006). Identification of insulin signaling elements in human beta-cells: Autocrine regulation of insulin gene expression.. Diabetes.

[7] Mallat S.G. (2013). Dual renin-angiotensin system inhibition for prevention of renal and cardiovascular events: do the latest trials challenge existing evidence?. Cardiovasc. Diabetol..

[8] Cheng Q., Leung P.S. (2011). An update on the islet renin–angiotensin system.. Peptides.

[9] Luther J.M., Brown N.J. (2011). The renin–angiotensin–aldosterone system and glucose homeostasis.. Trends Pharmacol. Sci..

[10] Graus-Nunes F., Souza-Mello V. (2019). The renin-angiotensin system as a target to solve the riddle of endocrine pancreas homeostasis.. Biomed. Pharmacother..

[11] Feng P., Wu Z., Liu H., Shen Y., Yao X., Li X., Shen Z. (2020). Electroacupuncture improved chronic cerebral hypoperfusion-induced anxiety-like behavior and memory impairments in spontaneously hypertensive rats by downregulating the ACE/Ang II/AT1R Axis and upregulating the ACE2/Ang-(1-7)/MasR Axis.. Neural Plast..

[12] Ali Q., Dhande I., Samuel P., Hussain T. (2016). Angiotensin type 2 receptor null mice express reduced levels of renal angiotensin II type 2 receptor/angiotensin (1-7)/Mas receptor and exhibit greater high-fat diet-induced kidney injury.. J. Renin Angiotensin Aldosterone Syst..

[13] Tiwari P, Tiwari V, Gupta S, Shukla S, Hanif K (2023). Activation of angiotensin-converting enzyme 2 protects against lipopolysaccharide-induced glial activation by modulating angiotensin-converting enzyme 2/angiotensin (1-7)/Mas Receptor Axis.. Mol. Neurobiol..

[14] Shoemaker R., AlSiraj Y., Chen J., Cassis L.A. (2019). Pancreatic AT1aR deficiency decreases insulin secretion in Obese C57BL/6 Mice.. Am. J. Hypertens..

[15] Leung K.K., Leung P.S. (2008). Effects of hyperglycemia on angiotensin II receptor type 1 expression and insulin secretion in an INS-1E pancreatic beta-cell line.. JOP.

[16] McMurray J.J., Holman R.R., Haffner S.M., Bethel M.A., Holzhauer B., Hua T.A., Belenkov Y., Boolell M., Buse J.B., Buckley B.M., Chacra A.R., Chiang F.T., Charbonnel B., Chow C.C., Davies M.J., Deedwania P., Diem P., Einhorn D., Fonseca V., Fulcher G.R., Gaciong Z., Gaztambide S., Giles T., Horton E., Ilkova H., Jenssen T., Kahn S.E., Krum H., Laakso M., Leiter L.A., Levitt N.S., Mareev V., Martinez F., Masson C., Mazzone T., Meaney E., Nesto R., Pan C., Prager R., Raptis S.A., Rutten G.E., Sandstroem H., Schaper F., Scheen A., Schmitz O., Sinay I., Soska V., Stender S., Tamás G., Tognoni G., Tuomilehto J., Villamil A.S., Vozár J., Califf R.M. (2010). Effect of valsartan on the incidence of diabetes and cardiovascular events.. N. Engl. J. Med..

[17] Li J., Zhu R., Liu Y., Yang J., Wang X., Geng L., Xu T., He J. (2021). Angiotensin-(1-7) improves islet function in a rat model of streptozotocin- induced diabetes mellitus by up-regulating the expression of Pdx1/Glut2.. Endocr. Metab. Immune Disord. Drug Targets.

[18] Bartel D.P. (2004). MicroRNAs.. Cell.

[19] Guay C., Roggli E., Nesca V., Jacovetti C., Regazzi R. (2011). Diabetes mellitus, a microRNA-related disease?. Transl. Res..

[20] Zhang A., Li D., Liu Y., Li J., Zhang Y., Zhang C.Y. (2018). Islet β cell: An endocrine cell secreting miRNAs.. Biochem. Biophys. Res. Commun..

[21] Ding Y., Zhong J., Wang Y., Xie W. (2020). Proteomic and microRNA omic profiles and potential mechanisms of dysfunction in pancreatic islet cells primed by inflammation.. Exp. Ther. Med..

[22] van de Bunt M., Gaulton K.J., Parts L., Moran I., Johnson P.R., Lindgren C.M., Ferrer J., Gloyn A.L., McCarthy M.I. (2013). The miRNA pro-file of human pancreatic islets and beta-cells and relationship to type 2 diabetes pathogenesis.. PLoS One.

[23] Eliasson L., Esguerra J.L.S. (2020). MicroRNA networks in pancreatic islet cells: Normal function and type 2 diabetes.. Diabetes.

[24] Obama T., Eguchi S. (2014). MicroRNA as a novel component of the tissue renin angiotensin system.. J. Mol. Cell. Cardiol..

[25] Arthurs A.L., Lumbers E.R., Pringle K.G. (2019). MicroRNA mimics that target the placental renin–angiotensin system inhibit trophoblast proliferation.. Mol. Hum. Reprod..

[26] Hagiwara S., McClelland A., Kantharidis P. (2013). MicroRNA in diabetic nephropathy: Renin angiotensin, aGE/RAGE, and oxidative stress pathway.. J. Diabetes Res..

[27] Wang T., Min X., Wang T., Cao Y., Liu J., Li J. (2015). MicroRNAs: A novel promising therapeutic target for cerebral ischemia/reperfusion injury?. Neural Regen. Res..

[28] Poy M.N., Eliasson L., Krutzfeldt J., Kuwajima S., Ma X., MacDonald P.E., Pfeffer S., Tuschl T., Rajewsky N., Rorsman P., Stoffel M. (2004). A pancreatic islet-specific microRNA regulates insulin secretion.. Nature.

[29] Eliasson L. (2017). The small RNA miR-375-a pancreatic islet abundant miRNA with multiple roles in endocrine beta cell function.. Mol. Cell. Endocrinol..

[30] Li X. (2014). miR-375, a microRNA related to diabetes.. Gene.

[31] Dumortier O., Fabris G., Pisani D.F., Casamento V., Gautier N., Hinault C., Lebrun P., Duranton C., Tauc M., Dalle S., Kerr-Conte J., Pattou F., Prentki M., Van Obberghen E. (2020). microRNA-375 regulates glucose metabolism-related signaling for insulin secretion.. J. Endocrinol..

[32] Hu S., Zhang M., Sun F., Ren L., He X., Hua J., Peng S. (2016). miR-375 controls porcine pancreatic stem cell fate by targeting 3-phosphoinositide-dependent protein kinase-1 (Pdk1).. Cell Prolif..

[33] Chen Z., Liu H., Yang H., Gao Y., Zhang G., Hu J. (2017). The long noncoding RNA, TINCR, functions as a competing endogenous RNA to regu-late PDK1 expression by sponging miR-375 in gastric cancer.. OncoTargets Ther..

[34] Jia-yuan X., Wei S., Fang-fang L., Zhi-jian D., Long-he C., Sen L. (2020). Corrigendum to “miR-375 inhibits the proliferation and invasion of nasopharyngeal carcinoma cells by suppressing PDK1”.. BioMed Res. Int..

[35] Wang J., Sun X. (2018). MicroRNA-375 inhibits the proliferation, migration and invasion of kidney cancer cells by triggering apoptosis and modulation of PDK1 expression.. Environ. Toxicol. Pharmacol..

[36] El Ouaamari A., Baroukh N., Martens G.A., Lebrun P., Pipeleers D., van Obberghen E. (2008). miR-375 targets 3′-phosphoinositide-dependent protein kinase-1 and regulates glucose-induced biological responses in pancreatic β-cells.. Diabetes.

[37] Li Y., Xu X., Liang Y., Liu S., Xiao H., Li F., Cheng H., Fu Z. (2010). miR-375 enhances palmitate-induced lipoapoptosis in insulin-secreting NIT-1 cells by repressing myotrophin (V1) protein expression.. Int. J. Clin. Exp. Pathol..

[38] Miyazaki J.I., Araki K., Yamato E., Ikegami H., Asano T., Shibasaki Y., Oka Y., Yamamura K.I. (1990). Establishment of a pancreatic beta cell line that retains glucose-inducible insulin secretion: special reference to expression of glucose transporter isoforms.. Endocrinology.

[39] Nakashima K., Kanda Y., Hirokawa Y., Kawasaki F., Matsuki M., Kaku K. (2009). MIN6 is not a pure beta cell line but a mixed cell line with other pancreatic endocrine hormones.. Endocr. J..

[40] Kovalski J.R., Bhaduri A., Zehnder A.M., Neela P.H., Che Y., Wozniak G.G., Khavari P.A. (2019). The functional proximal proteome of oncogenic ras includes mTORC2.. Mol. Cell.

[41] Yuan T., Lupse B., Maedler K., Ardestani A. (2018). mTORC2 Signaling: A path for pancreatic β Cell’s growth and function.. J. Mol. Biol..

[42] Kemp J.R., Unal H., Desnoyer R., Yue H., Bhatnagar A., Karnik S.S. (2014). Angiotensin II-regulated microRNA 483-3p directly targets multiple components of the renin–angiotensin system.. J. Mol. Cell. Cardiol..

[43] Yang M., Song J.J., Yang X.C., Zhong G.Z., Zhong J.C. (2022). MiRNA-122-5p inhibitor abolishes angiotensin II–mediated loss of autophagy and promotion of apoptosis in rat cardiofibroblasts by modulation of the apelin-AMPK-mTOR signaling.. In Vitro Cell. Dev. Biol. Anim..

[44] Arthurs A.L., Lumbers E.R., Delforce S.J., Mathe A., Morris B.J., Pringle K.G. (2019). The role of oxygen in regulating microRNAs in control of the placental renin–angiotensin system.. Mol. Hum. Reprod..

[45] Wu W.H., Hu C.P., Chen X.P., Zhang W.F., Li X.W., Xiong X.M., Li Y.J. (2011). MicroRNA-130a mediates proliferation of vascular smooth muscle cells in hypertension.. Am. J. Hypertens..

[46] Luo P., Zhang W.F., Qian Z.X., Xiao L.F., Wang H., Zhu T.T., Li F., Hu C.P., Zhang Z. (2016). MiR-590-5p-meidated LOX-1 upregulation promotes Angiotensin II-induced endothelial cell apoptosis.. Biochem. Biophys. Res. Commun..

[47] Feng H., Wu J., Chen P., Wang J., Deng Y., Zhu G., Xian J., Huang L., Ouyang W. (2019). MicroRNA‐375‐3p inhibitor suppresses angiotensin II‐induced cardiomyocyte hypertrophy by promoting lactate dehydrogenase B expression.. J. Cell. Physiol..

[48] Nathan G., Kredo-Russo S., Geiger T., Lenz A., Kaspi H., Hornstein E., Efrat S. (2015). MiR-375 promotes redifferentiation of adult human β cells expanded in vitro.. PLoS One.

[49] Gezginci-Oktayoglu S., Sancar S., Karatug-Kacar A., Bolkent S. (2021). miR‐375 induces adipogenesis through targeting Erk1 in pancreatic duct cells under the influence of sodium palmitate.. J. Cell. Physiol..

[50] Li C., Chen L., Zhao Y., Chen S., Fu L., Jiang Y., Gao S., Liu Z., Wang F., Zhu X., Rao J., Zhang J., Zhou X. (2017). Altered expression of miRNAs in the uterus from a letrozole-induced rat PCOS model.. Gene.

[51] Wei R., Yang J., Liu G., Gao M., Hou W., Zhang L., Gao H., Liu Y., Chen G., Hong T. (2013). Dynamic expression of microRNAs during the differentiation of human embryonic stem cells into insulin-producing cells.. Gene.

[52] Poy M.N., Hausser J., Trajkovski M., Braun M., Collins S., Rorsman P., Zavolan M., Stoffel M. (2009). miR-375 maintains normal pancreatic α- and β-cell mass.. Proc. Natl. Acad. Sci..

[53] Pudewell S., Lissy J., Nakhaeizadeh H., Mosaddeghzadeh N., Nakhaei-Rad S., Dvorsky R., Ahmadian M.R. (2022). New mechanistic insights into the RAS-SIN1 interaction at the membrane.. Front. Cell Dev. Biol..

[54] Jacinto E., Facchinetti V., Liu D., Soto N., Wei S., Jung S.Y., Huang Q., Qin J., Su B. (2006). SIN1/MIP1 maintains rictor-mTOR complex integ-rity and regulates Akt phosphorylation and substrate specificity.. Cell.

[55] Frias M.A., Thoreen C.C., Jaffe J.D., Schroder W., Sculley T., Carr S.A., Sabatini D.M. (2006). mSin1 is necessary for Akt/PKB phosphorylation, and its isoforms define three distinct mTORC2s.. Curr. Biol..

[56] Yang Q., Inoki K., Ikenoue T., Guan K.L. (2006). Identification of Sin1 as an essential TORC2 component required for complex formation and kinase activity.. Genes Dev..

[57] Moraitis D., Karanikou M., Liakou C., Dimas K., Tzimas G. (2014). SIN1, a critical component of the mTOR-Rictor complex, is overexpressed and associated with AKT activation in medullary and aggressive papillary thyroid carcinomas.. Surgery.

[58] Facchinetti V., Ouyang W., Wei H., Soto N., Lazorchak A., Gould C., Lowry C., Newton A.C., Mao Y., Miao R.Q., Sessa W.C., Qin J., Zhang P., Su B., Jacinto E. (2008). The mammalian target of rapamycin complex 2 controls folding and stability of Akt and protein kinase C.. EMBO J..

[59] García-Martínez J.M., Alessi D.R. (2008). mTOR complex 2 (mTORC2) controls hydrophobic motif phosphorylation and activation of serum- and glucocorticoid-induced protein kinase 1 (SGK1).. Biochem. J..

[60] Kim S.G., Sung J.Y., Kim J.R., Choi H.C. (2021). Fisetin-induced PTEN expression reverses cellular senescence by inhibiting the mTORC2-Akt Ser473 phosphorylation pathway in vascular smooth muscle cells.. Exp. Gerontol..

[61] Wang G., Liu M., Wang H., Yu S., Jiang Z., Sun J., Han K., Shen J., Zhu M., Lin Z., Jiang C., Guo M. (2016). Centrosomal protein of 55 regulates glucose metabolism, proliferation and apoptosis of glioma cells via the Akt/mTOR signaling pathway.. J. Cancer.

[62] Somanath P.R., Razorenova O.V., Chen J., Byzova T.V. (2006). Akt1 in endothelial cell and angiogenesis.. Cell Cycle.

[63] Jara M.A., Werneck-De-Castro J.P., Lubaczeuski C., Johnson J.D., Bernal-Mizrachi E. (2020). Pancreatic and duodenal homeobox-1 (PDX1) con-tributes to β-cell mass expansion and proliferation induced by Akt/PKB pathway.. Islets.

[64] da Costa R.M., Neves K.B., Mestriner F.L., Louzada-Junior P., Bruder-Nascimento T., Tostes R.C. (2016). TNF-α induces vascular insulin re-sistance via positive modulation of PTEN and decreased Akt/eNOS/NO signaling in high fat diet-fed mice.. Cardiovasc. Diabetol..

[65] Ždychová J., Komers R. (2005). Emerging role of Akt kinase/protein kinase B signaling in pathophysiology of diabetes and its complications.. Physiol. Res..

[66] Šrámek J., Němcová-Fürstová V., Kovář J. (2016). Kinase signaling in apoptosis induced by saturated fatty acids in pancreatic β-cells.. Int. J. Mol. Sci..

[67] Yao S., Zhang J., Zhan Y., Shi Y., Yu Y., Zheng L., Xu N., Luo G. (2020). Insulin resistance in apolipoprotein m knockout mice is mediated by the protein kinase Akt signaling pathway.. Endocr. Metab. Immune Disord. Drug Targets.

[68] Liu H., Zhang D., Zhou Y., Cui S. (2021). MicroRNA-7a inhibits Isl1 expression to regulate insulin secretion by targeting Raf1 and Mapkap1 in NIT-1 cells.. In Vitro Cell. Dev. Biol. Anim..

[69] Mukaida S., Evans B.A., Bengtsson T., Hutchinson D.S., Sato M. (2017). Adrenoceptors promote glucose uptake into adipocytes and muscle by an insulin-independent signaling pathway involving mechanistic target of rapamycin complex 2.. Pharmacol. Res..

[70] Yang J., Waldron R.T., Su H.Y., Moro A., Chang H.H., Eibl G., Ferreri K., Kandeel F.R., Lugea A., Li L., Pandol S.J. (2016). Insulin promotes proliferation and fibrosing responses in activated pancreatic stellate cells.. Am. J. Physiol. Gastrointest. Liver Physiol..

