# Supplementary material for: Ang II Controls the Expression of Mapkap1 by miR-375 and Affects the Function of Islet β Cells
Source: Endocr Metab Immune Disord Drug Targets. 2023 Jul 19;23(9):1186–200. doi: 10.2174/1871530323666230206121715 (PMC10514520; doi:10.2174/1871530323666230206121715)
Supplement: Supplementary file 1 [file EMIDDT-23-1186_SD1.pdf]

Supplementary Material

Ang II Controls the Expression of Mapkap1 by miR-375 and Affects the Function of Islet β Cells

Xiuhong Lin[1,*], Lin Cheng[2,*], Yan Wan[2], Yuerong Yan[2], Zhuo Zhang[2], Xiaohui Li[2], Jiayun Wu[2], Xiaoyi Wang[2] and Mingtong Xu[2]

[1]Department of Clinical Nutrition, Sun Yat-sen Memorial Hospital, Sun Yat-sen University, No. 107 Yanjiangxi Road, Guangzhou, Guangdong, People's Republic of China, 510120, China, [2]Department of Endocrinology, Sun Yat-sen Memorial Hospital, Sun Yat-sen University, No. 107 Yanjiangxi Road, Guangzhou, Guangdong, People's Republic of China, 510120, China

A

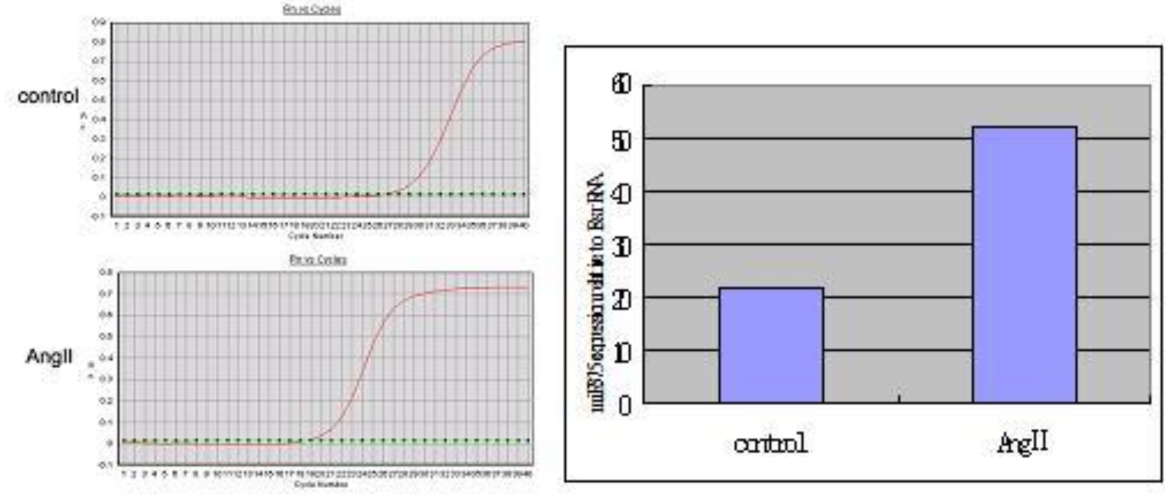

B

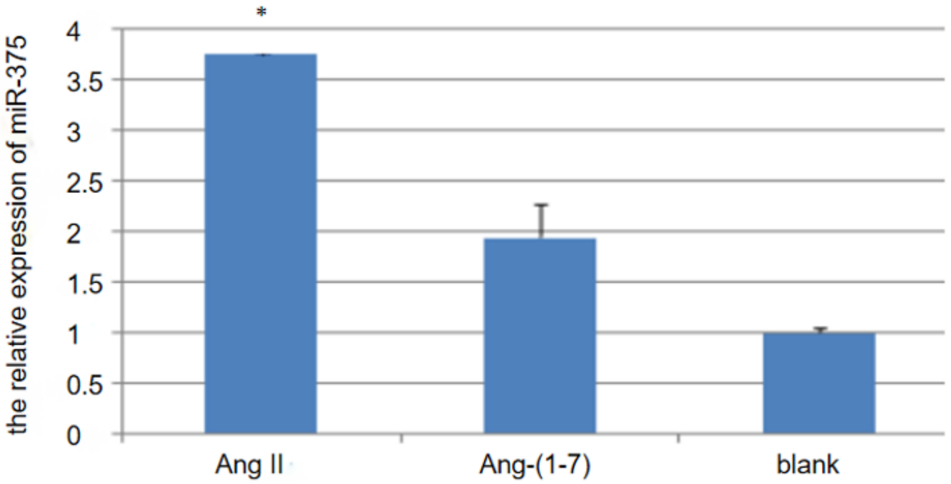

Fig. (S1). Preliminary experimental results, miRNA real-time PCR results. (A) real - time PCR detect the change of miR-375 after Ang II acting on MIN6 cells, the results showed that the expression levels of miR-375 significantly increased in comparison with the control group. (B) The expression of miR-375 under RAS was detected by Real-time PCR.

Abbreviations: *Ang II: Angiotensin II; Ang-(1-7): Angiotensin-(1-7). *P < 0.05 vs blank.*

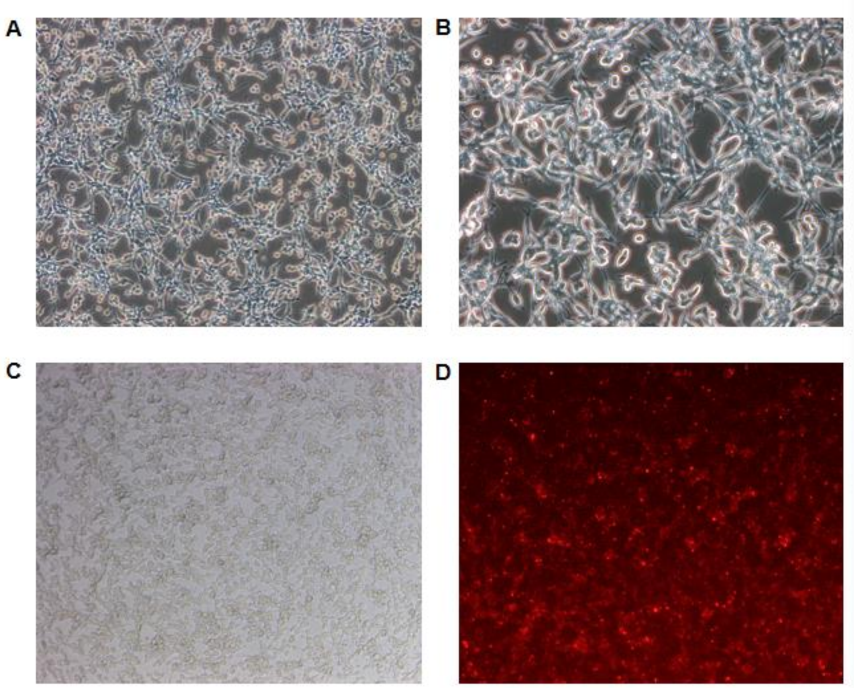

Fig. (S2). Observation of MIN6 under ordinary microscope (100×) (A), (200×) (B). Normal cultured MIN6 cells adhered to the wall, and the individual cells were polygonal and distributed in clusters. (C) Observation of MIN6 under fluorescence microscope (100×); (D) Transfection efficiency observed by green fluorescence under fluorescence microscope (100×). After transfection of NC-CY3 (negative miRNA labeled with red fluorescence) for 24 hours, green fluorescence was observed under inverted fluorescence microscope, and the transfection efficiency was about 80%-90%.

A

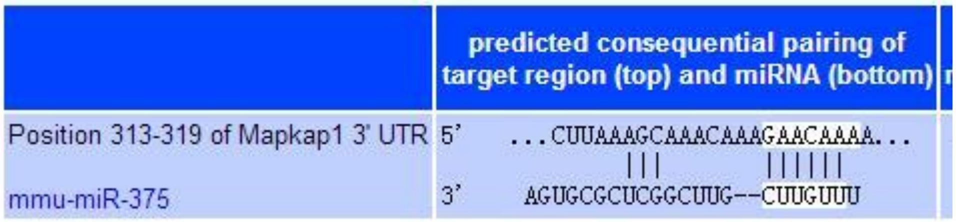

B

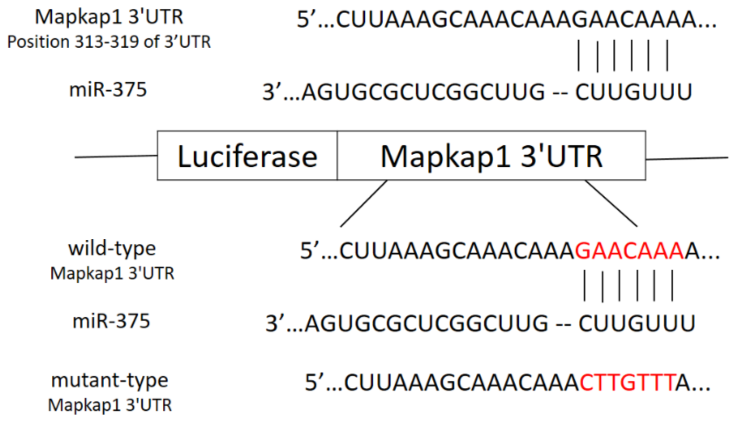

Fig. (S3). Detection of target genes. (**A**) Bioinformatics software predicted the binding site of Mapkap1 to miR-375. (**B**) Luciferase reporter assay: miR-375 directly binds Mapkap1.

Table S1. Primer sequence for PCR

**Gene**	**Sequence**
miR-375	F: 5’- CGCGCTTTGTTCGTTCGGCTC - 3’<br>R:5’- ATCCAGTGCAGGGTCCGAGG - 3’
U6	F: 5’-CTCGCTTCGGCAGCACA-3’<br>R: 5’- AACGCTTCACGAATTTGCGT - 3’
Mapkap1	F: 5’- TGTCTCCAAGTGGTGCTGAG –3’<br>R: 5’- AGAGAGGCAGGCTGACCATA -3’
β-actin	F: 5’- CATCCGTAAAGACCTCTATGCCAAC -3’<br>R: 5’- ATGGAGCCACCGATCCACA -3’
